# Regular Flatulence Patterns Among Community-Dwelling Individuals in Australia

**DOI:** 10.1001/jamanetworkopen.2026.15637

**Published:** 2026-05-29

**Authors:** Emily Brindal, Danielle Baird

**Affiliations:** 1 Health & Biosecurity, Commonwealth Scientific and Industrial Research Organisation, Kaurna Land, Adelaide, South Australia, Australia

## Abstract

This cross-sectional study examines daily flatulence patterns reported by individuals in Australia.

## Introduction

Flatulence is defined as the act of passing flatus through the anus, with some definitions incorporating the experience of excessive gas. Flatulence research has focused largely on disease and symptomology,^1,2^ yet intestinal gas is part of healthy digestion. The value of flatus as an indicator of digestive health has best utility when “excessive” is clearly defined. Nutrition textbooks suggest a normal range of 5 to 20 outputs per day based on fiber intake. However, most investigators have shied away from asking how frequently the average person passes gas. The aim of our study was to capture the flatulence behavior of free living community-dwelling individuals in Australia using noninvasive methods.

## Methods

This cross-sectional study was approved by the Low Risk Human Research Ethics Committee of the Commonwealth Scientific and Industrial Research Organisation and reported based on STROBE guidelines. Data were recorded into a purpose-designed mobile phone application (Chart Your Fart) by participants who logged their flatus passages in real time, consistent with experience sampling methods.^3^ Participants were recruited using nonprobability sampling methods and invited to take part through a large national communication campaign targeting various audiences and media. Participants provided digital informed consent through the app. Inclusion criteria were as follows: access to compatible device (Android and iOS), residing in Australia, and age 14 years or older. Exclusion criteria included self-reported large changes to diet and failing to provide digital consent during registration. Consenting participants were instructed to enter each passage as close as possible to its discharge over at least 2 weekdays and 1 weekend day. Time of event could be retrospectively edited. The app was designed to make recording simple and discreet. Participants could enter, review, and chart only their own entries to minimize socially desirable reporting. Data collection occurred from campaign launch (November 2024) to February 2025.

The primary outcome considered was total flatus per day (fls/d), which was averaged within person and summarized through means and medians. Kruskal-Wallis nonparametric tests, performed in SPSS, version 29 (IBM Inc), were used to assess differences in fls/d between gender and age groups. Significant main effects (*P* < .05, 2-sided) were assessed using Bonferroni-adjusted pairwise comparisons. Total flatus entries were summed to explore patterns across the day.

## Results

Consent was provided by 19 004 individuals. After screening, the final sample included 6416 individuals who entered 360 192 outputs (8.3% retrospectively adjusted). The oldest age group was undersampled; otherwise, the sample broadly represented the Australian population^4^ (4925 [76.8%] aged 26 to 65 years; 3255 [50.7%] female; 1959 [30.5%] with bachelor’s degrees; 4740 [73.9%] from eastern states) (Table).

**Table.** Average Daily Flatus Releases Across Sample (N = 6416)^a^

Measure	Sample, No. (%)	Australian population, %^b^	Flatus per day		χ^2^	*P* value
			Mean (SD)	Median (IQR)		
Total	6416 (100)	100	5.0 (3.8)	3.8 (2.5-6.2)	NA	NA
Gender						
Female	3255 (50.7)	50.7	4.8 (3.6)	3.8 (2.4-6.0)	12.98	.002^c^
Male	3015 (47.0)	49.3	5.2 (3.9)	4.0 (2.5-6.5)		
Other^d^	146 (<1.0)	NA	5.4 (4.8)	4.0 (2.3-7.3)		
Age group, y						
14-25	852 (13.3)	17.7	4.4 (3.4)	3.3 (2.2-5.3)	47.38	<.001^e^
26-45	2444 (38.1)	33.9	5.2 (3.8)	4.0 (2.6-6.5)		
46-65	2481 (38.7)	29.2	5.0 (3.9)	4.0 (2.5-6.3)		
≥66	639 (10.0)	19.2	4.8 (3.7)	3.7 (2.4-5.8)		

Abbreviation: NA, not applicable.

^a^ Presented by gender and age groups based on within person average. Compared using Kruskal-Wallis nonparametric tests.

^b^ Based on Australian Bureau of Statistics, Population clock and pyramid, including only individuals 14 years and over (n = 21 335 131) and 2021 Census data, accessed April 2026.

^c^ Significant pairwise comparisons with Bonferroni adjustments (*P* = .001): female vs male.

^d^ Includes nonbinary (n = 112), other (n = 10), and preferred not to say (n = 24).

^e^ Significant pairwise comparisons with Bonferroni adjustments: 14-25 vs 26-45 years (*P* < .001); 14-25 vs 46-65 years (*P* < .001); 14-25 vs ≥66 years (*P* = .02).

Participants recorded entries for a mean of 10 days (range, 3-97 days). The sample mean was 5.0 fls/d (SD, 3.8 fls/d), with a median of 3.8 fls/d (IQR, 2.5-6.2 fls/d); 5085 (79.3%) recorded between 2 and 7 fls/d. Males recorded higher activity than females. The youngest age group reported fewer daily releases compared with all other age groups (Table).

Across-day variation showed a gradual increase with a peak between 6 and 10 pm (Figure). This overlapped with general population total energy and fiber intake,^5^ with a notable dip in flatulence recordings in the middle of the day.

**Figure.**
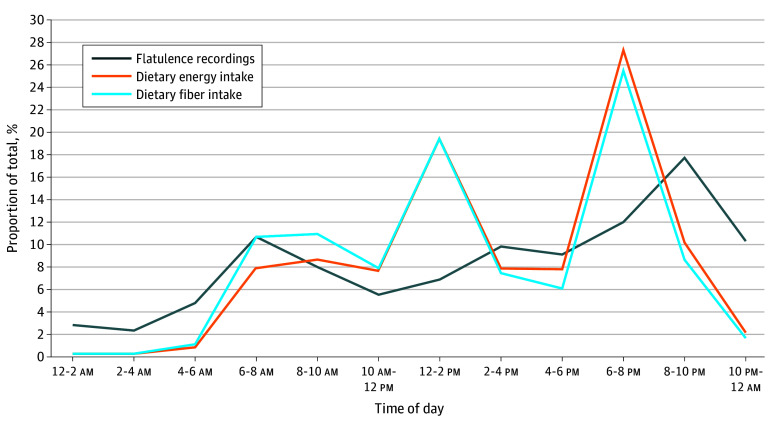
Flatulence Activity and Dietary Energy and Fiber Intake Flatulence activity across the day is presented as a proportion of total flatus recorded. Population total dietary energy and fiber intake data are presented as a proportion of adult total intake based on national data.

## Discussion

Our data are, to our knowledge, the first to describe real-time flatulence habits in a large, general population. In terms of range, observed data suggest good consistency with other methods, including retrospective frequency reports in a large US sample of individuals experiencing gas or bloating (n = 16 537),^6^ a small laboratory study that collected a median of 8 emissions throughout 24 hours,^7^ and even Benjamin Franklin’s personal account of “discharging wind from bowels” 7 times a day.^8^ One small sample (n = 25) using a physical diary recorded higher mean rates over 1 week (10 fls/d).^9^ Differences across age groups were also consistent with a previous report.^6^

Limitations of this study include failing to quantify emissions made while asleep due to reliance on self-report. Devices inserted into the anus to capture intestinal gas provide rigorous observation of flatulence production,^7^ but production is unlikely to be perfectly correlated with output, given conscious control over release and associated sociocultural standards. Furthermore, perception of excess relies on patient self-report. Nonetheless, self-report may also partially explain the observed gender differences. Greater efficiency in expulsion could also confound frequency data, with large variation in individual volume previously observed (33-125 mL/flatus).^7^ Lack of entry was not differentiated from lack of flatus passage; participants could not indicate a null-flatus day. Null-flatus days may occur,^6^ which, if uncaptured, may inflate our observations. Despite reasonable representation of the Australian population, broader effects of selection bias (eg, particular interest in gastrointestinal health or technological savviness) were not assessed.

Our study provides a good indication of regular flatulence habits and a starting point for conversations about excess. High participation and sustained engagement also indicate flatulence is an area of interest in the population and warrants greater discussion.

## Notes

Supplement.
Data Sharing Statement
