# Supplementary material for: Regular Flatulence Patterns Among Community-Dwelling Individuals in Australia
Source: JAMA Netw Open. 2026 May 29;9(5):e2615637. doi: 10.1001/jamanetworkopen.2026.15637 (PMC13221684; doi:10.1001/jamanetworkopen.2026.15637)
Supplement: Supplement. — Data Sharing Statement [file jamanetwopen-e2615637-s001.pdf]

Data Sharing Statement

Brindal. Regular Flatulence Patterns Among Community-Dwelling Individuals in Australia. *JAMA Netw Open*. Published May 29, 2026. doi:10.1001/jamanetworkopen.2026.15637

Data
Data available: Yes
Data types: Deidentified participant data
How to access data: Email corresponding author at emily.brindal@csiro.au.
When available: With publication

Supporting Documents
Document types: None

Additional Information
Who can access the data: De-identified data will be made available for the complete dataset with ethical approval.
Types of analyses: Anything with an ethically approved research objective.
Mechanisms of data availability: With internal ethical approval and a data sharing agreement, data will be shared with investigator support as needed.
